# Vimentin Association with Nuclear Grooves in Normal MEF 3T3 Cells

**DOI:** 10.3390/ijms21207478

**Published:** 2020-10-10

**Authors:** Karolina Feliksiak, Tomasz Witko, Daria Solarz, Maciej Guzik, Zenon Rajfur

**Affiliations:** 1 Faculty of Physics, Astronomy and Applied Computer Science, Jagiellonian University, 30-348 Kraków, Poland; karolina.feliksiak89@gmail.com (K.F.); daria.solarz@doctoral.uj.edu.pl (D.S.); 2 Jerzy Haber Institute of Catalysis and Surface Chemistry Polish Academy of Sciences, 30-239 Kraków, Poland; tomasz.witko@ikifp.edu.pl

**Keywords:** cytoskeleton, intermediate filaments, vimentin, grooved nuclei, polyhydroxyoctanoate (PHO), polylactide (PLA), fibroblasts, cancer

## Abstract

Vimentin, an intermediate filament protein present in leukocytes, blood vessel endothelial cells, and multiple mesenchymal cells, such as mouse embryonic fibroblasts (MEF 3T3), is crucial for various cellular processes, as well as for maintaining the integrity and durability (stability) of the cell cytoskeleton. Vimentin intermediate filaments (VIFs) adhere tightly to the nucleus and spread to the lamellipodium and tail of the cell, serving as a connector between the nucleus, and the cell’s edges, especially in terms of transferring mechanical signals throughout the cell. How these signals are transmitted exactly remains under investigation. In the presented work, we propose that vimentin is involved in that transition by influencing the shape of the nucleus through the formation of nuclear blebs and grooves, as demonstrated by microscopic observations of healthy MEF (3T3) cells. Grooved, or “coffee beans” nuclei, have, to date, been noticed in several healthy cells; however, these structures are especially frequent in cancer cells—they serve as a significant marker for recognition of multiple cancers. We observed 288 MEF3T3 cells cultured on polyhydroxyoctanoate (PHO), polylactide (PLA), and glass, and we identified grooves, coaligned with vimentin fibers in the nuclei of 47% of cells cultured on PHO, 50% of cells on glass, and 59% of cells growing on PLA. We also observed nuclear blebs and associated their occurrence with the type of substrate used for cell culture. We propose that the higher rate of blebs in the nuclei of cells, cultured on PLA, is related to the microenvironmental features of the substrate, pH in particular.

## 1. Introduction

Vimentin is an intermediate filament protein present in a variety of endothelial and mesenchymal cells. It is mostly known for protecting the cell as a whole, and its inner organelles separately [1], and also for ensuring cell cytoskeleton stability [2,3]. Its role is not limited to shielding the cell’s organelles, though, as it is also involved in active mechanotransduction of the signals from the exterior of the cell to its core [4]. Especially interesting are the dependencies between the cell nucleus and vimentin intermediate filaments (VIFs), as recent evidence indicates the importance of vimentin in regulating nuclear mechanics and shape [5].

Grooved nuclei, often called “coffee-beans nuclei” [6] are diagnostic markers of cancer, common for benign and malignant tumors, as papillary thyroid carcinoma, melanoma, hepatocellular carcinoma, or pituitary adenoma [7,8,9]. It is hypothesized that these indentations might be the inclusions of the nuclear envelope, caused by an inward push of larger amounts of cytoplasmic organelles in the space-restricted cytoplasmic areas [9]. The invaginations in the nuclei are usually referred to the pathological feature of cancer cells and are identified as longitudinal nuclear membrane folds along the long axis of the nucleus. The shape of these nuclei resembles coffee beans, yet the pathogenesis beyond the presence of these structures is not known. It has been shown that neither of the major structural proteins of the nuclear envelope, such as lamin A, B, B1, and C, nor lamin B receptor or emerin, are involved in the formation of the grooves [10].

It has also been proved that the grooves, present in some plant cells [11], were coaligned with microfilaments (F-actin fibers). The same study has shown, however, that disruption of actin did not influence the presence of the grooves [11]. The final observations suggest that polymerization of MTs is crucial for creating small but frequent fluctuations of the nuclear envelope, yet to obtain the grooves, MTs need to polymerize into bundles, which is dependent on Dynein. Within the absence of inner nuclear membrane protein—Kugelkern (Kuk), the invaginations initiated by MTs were not identified [12]. This indicates that another cytoskeleton component might participate in creating the grooves. In this work, we show that the nuclei grooves in 3T3 mouse embryonic fibroblast cells coalign with an intermediate filament cytoskeleton structural protein—vimentin. 

Vimentin has in the past been considered as a stable protein, maintaining cell stiffness and protecting it from mechanical damage [13]. Nowadays, it is known that vimentin is a dynamic structure that associates with the nuclear envelope proteins directly through LINC complex (linker of the nucleoskeleton to the cytoskeleton), or indirectly by the microfilaments (actin filaments) or microtubules that cooperate with the LINC complex [14]. Together with keratin, desmin, and GFAP, vimentin intermediate filaments are involved in nuclear movement and positioning, and recent evidence suggests that the levels of vimentin in cells influence the organization of chromatin and nucleus rigidity [15]. On the other hand, although direct and indirect contacts between the nucleus and the cytoskeleton influence the isometric tension of the nucleus, and are suspected to be core determinants of its shape, there are reports that bind the other factors—namely BRG1–chromatin remodeling enzyme ATPase—with the ultimate nucleus shape [16]. Another thesis referring to the chromatin–vimentin dependencies, shows that removal of the vimentin tail domain has a significant impact on proper mitotic progression, and can lead to impaired division [17].

During our vimentin cytoskeleton studies, we identified that in a fraction of mouse embryonic fibroblast cells, vimentin IFs form thick bundles, mostly above the nucleus, that are coaligned with the nuclei grooves. It has been reported that 3T3 cells have nuclear invaginations, containing F-actin [18], and that vimentin perinuclear rings influence the grooves of the human pancreatic cancer cells [19]. There also is evidence that the invaginated nuclei have been identified for healthy muscle cells [20] and that the indentations filled with actin are related to the degree of cell de-differentiation in human mammary epithelial tumor cells. It has also been reported that the nuclei with invaginations relate to the higher nuclear activity [21].

The nuclear deformations are not only grooves and invaginations but also changes in the shape of the nucleus (i.e., narrowing one end of the nucleus) or the presence of nuclear blebs. Blebbing is especially connected to cellular migration through small channels, and, in association with vimentin, there are reports which indicate that the absence of VIFs influences the increase in the number of nuclear blebs in migrating cells, and point out VIF’s protective function towards the nucleus [5,22]. This leads to the further investigation of the influence of the vimentin’s presence in the cells on the nuclear lamina. It has been shown that in the vimentin-depleted human SW-13 cells, the nucleus shape is affected by multiple invaginations, whereas vim+ SW-13 cells have much more regular and smooth nuclei [23]. Equally important in establishing the role of vimentin in nuclei shape could be the time point of the adhesion to the substrate. During the first steps of adhesion, vimentin forms ball-shaped structures around the nucleus, and surrounds it in the form of rings. Although these rings disappear in the 6–12 h of adhesion, there are reports confirming that the rings deform the nuclei and might lead to their rupture [24].

Since all these reports bring up other ideas in the final establishment of the processes standing behind the nucleus mechanics, we decided to check the influence of the microenvironment on the presence of the nuclei invaginations and blebs. To explore this further, we stained vimentin, microtubules, actin, and nucleus, and used confocal microscopy imaging for mouse embryonic fibroblast 3T3 cells, cultured on glass, and two substrates of high potential use in medicine: polylactide (PLA) [25,26] and polyhydroxyoctanoate (PHO)—a polymer of biological origin [27,28].

## 2. Results

### 2.1. Vimentin Bundles Associated with Nuclear Grooves of the Cells

**Figure 1:**
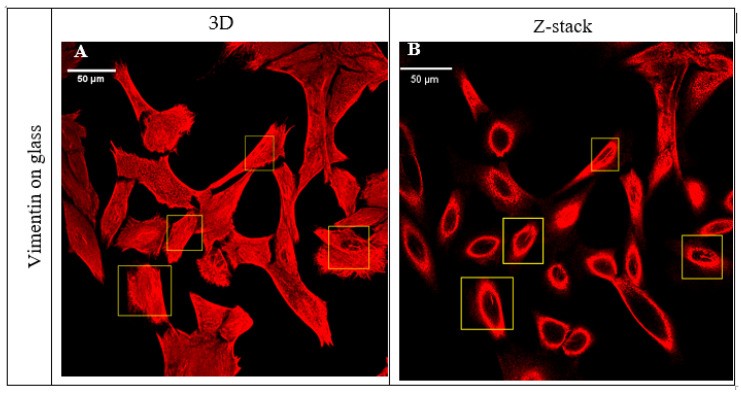
Vimentin in MEF 3T3 cells cultured on glass. The regions marked with the yellow frame present the cells in which there could be observed a thicker bundle of vimentin fibers above the nucleus. Pictures present overall projection (**A**) and single Z-section image (**B**).

In this work, we investigated the morphology of vimentin cytoskeleton in mouse embryonic fibroblast cells (MEF 3T3) cultured on glass and two materials of high potential application in biomedicine and tissue engineering—polyhydroxyoctanoate (PHO) and polylactide (PLA). We managed to observe thicker bundles of vimentin fibers in a fraction of the cells cultured on the glass, as shown in the Figure 1. 

To investigate this further, we stained the cells’ nucleus, vimentin, microtubules, and microfilaments, and scanned cells separately, using a confocal microscope. It was possible to identify the bundles of vimentin that were coaligned with either microtubules or actin microfilaments.

**Figure 2:**
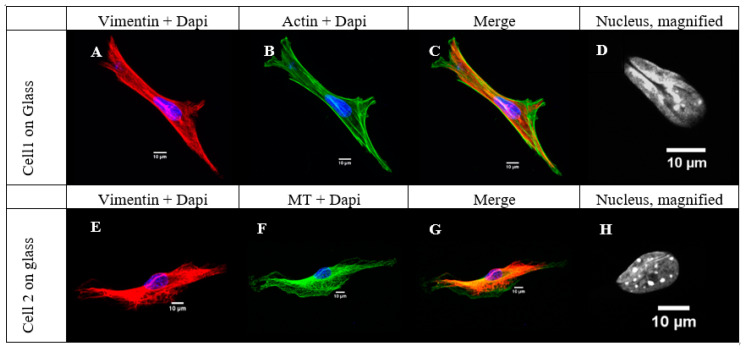
Examples of two cells cultured on glass, stained for vimentin, microfilaments, and nucleus (Cell1, panels (**A**–**D**)) and for vimentin, microtubules (MT), and nucleus (Cell2, panels (**E**–**H**)). Nucleus presented in magnification—with a clear groove, characteristic of “coffee-beans” nuclei.

Figure 2 shows an example of two cells with the “coffee bean” nucleus type. These cells were cultured on glass, and the nuclei with the grooves were identifiable at sight, as the thick vimentin bundle is coaligned with actin filaments (Cell 1) or microtubules (Cell 2), and passes precisely above nucleus in its central area, which is easily noticeable. After a deeper investigation of the nuclei for the cells cultured on different substrates, we observed that the grooves appeared not only in the central part of the nucleus but on the sides and in the bottom part of the nuclear area as well. These grooves are often not immediately visible with wide-field microscopy observation; however, our Z-stack images of single cells allowed us to observe the 3D structure and cross-section pictures of the nuclei. As a result, we were able to identify side and bottom irregular grooves in multiple cells for all the substrates.

**Figure 3:**
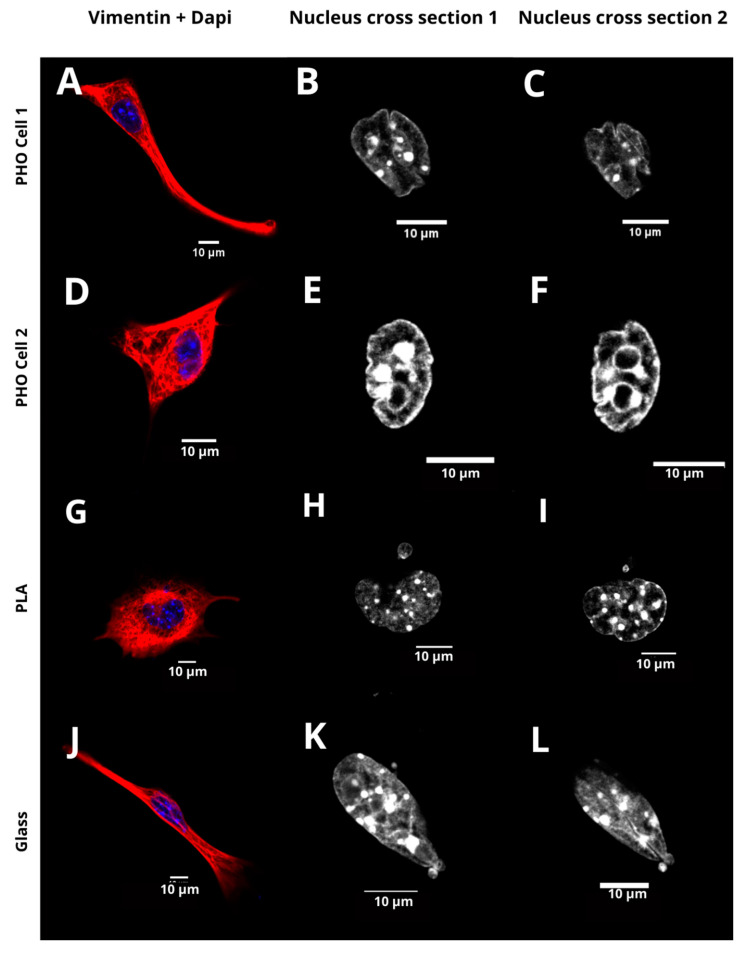
Examples of grooves and deformations of nuclei in MEF 3T3 cells cultured on glass (**J**–**L**), PHO (polyhydroxyoctanoate) (**A**–**F**), and PLA (polylactide) substrates (**G**–**I**). Two cross sections were selected for each nucleus.

In Figure 3, we present the examples of identified grooves and deformations of nuclei for MEF 3T3 cells cultured on PHO, PLA, and glass. Interestingly, vimentin fibers not only are present and fill the grooves and invaginations but also associate with differences in the terminal shape of the nucleus. Figure 3 shows 2 cells cultured on PHO and one cell on PLA for which the grooves are not located in the upper, central area of the nucleus (as for the “coffee-bean” nuclei) but on the side parts of the nuclei. The invaginations are irregular, with a different depth and size, but all are colocalized with running vimentin bundles. Moreover, the cell cultured on glass not only has longitudinal grooves in the upper central region of the nucleus, but also the nucleus is clearly narrower at one end than at the other. It is also noticeable that the nuclear blebs are formed in two cells presented on PLA and glass (Figure 3H,K,L) which occurs when significant mechanical stress is put on the cells [5]. The study focused on identifying all the nuclei that had any type of grooves—either the “coffee-bean” nuclei or the irregular invaginations, presented in Figure 3. Altogether, 288 cells on 3 different substrates were scanned and evaluated in terms of the presence of grooved nuclei. For each substrate, we prepared 5 samples, which provided the opportunity to scan 15 to 25 cells per sample. The numbers of the cells with nuclear grooves observed turned out to be statistically non-significant, per *χ*^2^ test, which gave a result of *χ*^2^(2) = 2.378, *p* > 0.05. It was identified that the grooved nuclei were present in 47% of cells cultured on PHO and 50% of cells on glass. For cells cultured on PLA, the amount of the cells with nuclear grooves was slightly higher—59% of the cells. This suggests the influence of the substrate on the presence of the invaginations, yet the cause of creating the grooves remains unknown.

**Figure 4:**
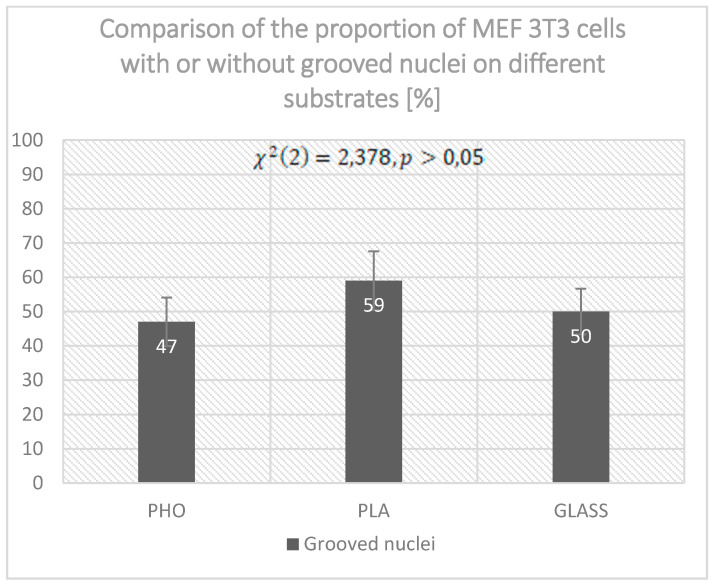
Comparison of the amount of the cells with or without grooved nuclei for cultures grown on PHO, PLA, and glass.

As presented in Figure 4, the cells with grooved nuclei that were cultured on PHO—were only 47%. For PLA—59%, and for glass—50% of the cells had nuclear grooves. This was not statistically significant.

**Figure 5:**
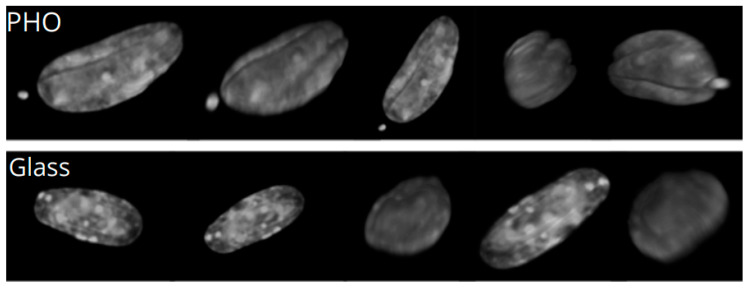
Example of 3D nucleus reconstruction for selected cells with grooves.

An example of 3D nucleus reconstruction for selected cells with grooves is presented below to visualize the exact location of such formations for 2 cells cultured on PHO and glass. As shown in Figure 5, the nuclear grooves can be difficult to observe. The 3D reconstruction allowed us to identify and confirm the presence of the invaginations in the cell nuclei, yet it would be insufficient for further numeric measurements—i.e., evaluation of length or depth.

### 2.2. Nuclear Blebs Associated with the Type of the Substrate

The analysis of the cells cultured on different substrates led to the observations of nuclear blebs that occurred for MEF 3T3 cells when cultured on all of the substrates used. For all of the cells, despite the substrate on which they were cultured, we could observe a number of the nuclear blebs. For the PLA, the number of the nuclei with blebs turned out to be statistically important, which was evaluated by the *χ*^2^ test. 

**Figure 6:**
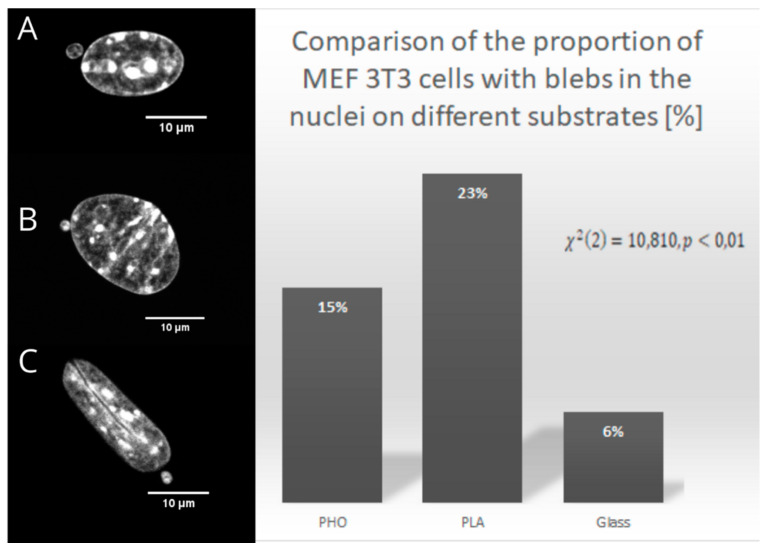
Examples of nuclear blebs for cells cultured on PHO (**A**), PLA (**B**), and glass (**C**). The graph presents the percentage of the cells in groups cultured on PHO, PLA, and Glass.

Figure 6 presents examples of nuclear blebs for the cells cultured on different substrates. Nuclear bleb occurrence might be a matter of changing the patterns of expression of specific isoforms of lamin, but also can appear spontaneously during cell migration through narrow spaces [5,22]. Analysis of the cells cultured on PLA, PHO, and glass showed that 23% of the cells on PLA have nuclear blebs. For cells cultured on PHO, there is 15% blebbed nuclei, whereas for glass, only 6% of the cells have blebs in the nucleus. To check if the number of nuclei with grooves might be related to the substrate used, we performed a *χ*^2^ test, which showed that there are no dependencies between the number of the grooved nuclei and the substrate. (*χ*^2^_0.05_ = 2.378). Analysis of the number of nuclei that were narrower at one end brought similar conclusions: *χ*^2^_0.05_ = 4.456. However, considering the nuclei with blebs, after careful analysis and performing *χ*^2^ test, the results indicated that the occurrence of the blebs might be dependent on the substrate used for cell culturing (*χ*^2^_0.01_ =10.810). Since the *χ*^2^ test confirms statistical significance, the influence of the substrate on the nuclei deformation might be confirmed. The highest number of cells with nuclear blebs on PLA might be correlated to the fact that PLA easily hydrolyses to the culture medium, influencing its pH.

### 2.3. Vimentin Sinks into the Grooves Deeper than Microtubules or Microfilaments

To check how vimentin correlates with actin and microtubules in associating the nuclear grooves, we stained two groups of MEF 3T3 cells for vimentin and nucleus, and one of those for actin filaments and the second one for microtubules. We scanned the cells using the same technique, and we obtained the cross-sections of the nuclei that visualize how deep the vimentin penetrates the nuclear grooves in comparison to actin filaments and microtubules. We investigated cells grown on all three substrates (PHO, PLA, and glass) and identified that in each case, vimentin was aligned closer to the nucleus than either f-actin or microtubules.

**Figure 7:**
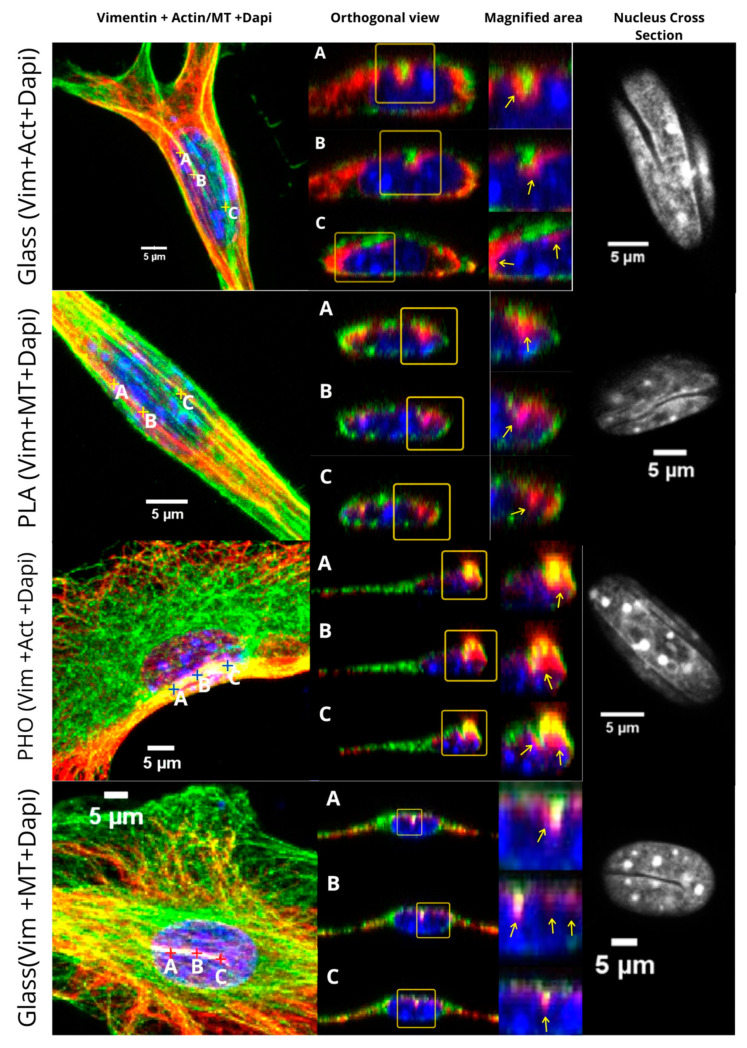
Comparison of grooves for selected MEF 3T3 cells, cultured on Glass, PHO, and PLA. Cells stained for actin or microtubules (green), vimentin (red), and Dapi (blue). Red, yellow and blue “+” signs (marked as **A**, **B**, **C** per each sub image) indicate the spots chosen for cross-section images. Yellow frames indicate the magnified areas of **A**–**C** spots, yellow arrows indicate the regions of interest.

In Figure 7, we present the examples of the cells with the grooved nuclei, cultured on different substrates and stained for either f-actin or microtubules accompanied with vimentin and Dapi. For all cells, we present the magnified area of the nucleus with the bundles of vimentin and actin or vimentin and microtubules that are present in the invaginations in the nucleus. The cross-section of the nuclei shows the invaginations, whereas the orthogonal view presents the cross section of all cytoskeletal elements, where we can observe the vimentin and MT/f-actin arrangement in the grooves. The mechanisms behind these processes remain under investigation.

## 3. Discussion

Grooved nuclei, although present in many types of healthy cells, are characteristic of multiple tumors, such as the pseudopapillary tumor of the pancreas [29], and serve as a marker for diagnosis of different types of cancers. It is known that for some cells, nuclear invaginations are dependent on the assembly of microtubules by a chromatin-bound factor, dappa2 (developmental pluripotency associated factor 2) [30]. We investigated 288 nuclei of MEF 3T3 cells and found that not only is vimentin filling the spaces created by the nuclear grooves, whose creation was, to date, associated with microtubules, but also that it reaches the deeper areas of the invaginations towards the interior of the nucleus. Since it is known that vimentin protects the nucleus from the mechanical forces, and supports its integrity [5], the question is if vimentin causes the creation of the grooves during movement of cells and their migration, or if the grooves are initially created by microtubules, and vimentin only serves as a protection against further nuclei deformation. Regardless of the size, depth, or location of the identified grooves, it appears that vimentin bundles reach deeper into the nuclear area than either actin or microtubules. Although the absence of actin does not influence the formation of grooves [11], for microtubules, the case is different. It has been shown that microtubules influence the shape of the nuclear envelope, and also that they have an impact on mechanical regulation of chromatin dynamics [12]. Since vimentin coaligns with microtubule bundles and is involved in filling the grooves, and sinks in the invaginations deeper, this suggests that it might be required for these structures to be present. Intermediate filaments are generally known for their affinity to histones [31] and for their protective functions towards cell nuclei and cellular integrity [5], yet the question might arise whether the vimentin bundles cooperate with microtubules in creating the grooves and deepening the fluctuations in the nuclear envelope, or do they act as a protective layer against microtubules or other factors that groove the nucleus? Does vimentin only appear in the deeper regions of the invaginations to protect the nucleus from further damage that is initially induced by the microtubules? It has been hypothesized that oligomers of intermediate filaments are able to displace octamers of histones from nucleosomes, in transcription initiation and elongation, and deposit these octamers on their coiled-coil domains [32].

Another case is considering nuclear grooves that were, to date, mostly seen as a feature of cancer cells, as a common characteristic of the cells exposed to certain conditions. The grooves of different kinds were identified in the nuclei of approximately half of the 3T3 cells that were cultured on PHO, PLA, and glass, with a slightly higher rate of these for PLA substrate (58%). This could suggest that “coffee-bean” nuclei might not be the feature of cancer cells per se, but rather a feature of cells with a strong mesenchymal phenotype. The presence of the cells with nuclear grooves and blebs on PLA substrate also suggests the influence of the microenvironment on the occurrence of these structures. As it is known that the extracellular acidic pH is related in general to cancer cells [33], it could be taken into consideration that the kind of a substrate might have an influence on the presence of the blebs. Cells grown on PLA presented a higher number of nuclei with blebs, and we suggest that this might be due to the fact that during cell growth, PLA was hydrolyzed to the medium in a form of lactic acid (pK_a_ = 3.86) [34]. This, in consequence, could influence the pH of the medium, causing it to become more acidic. PLA is known to easily degrade through the process of hydrolysis. Although PHO is slightly more resistant to that process, it also degrades to the cellular medium, influencing its pH [35,36,37,38]. However, since the occurrence of the grooves is statistically non-significant in terms of the type of the substrate used for cell culture, further experiments should focus on investigating the grooves themselves, as well as the association of their occurrence with cytoskeleton dependencies. The occurrence of the blebs, however, might be further considered in terms of the external, microenvironmental conditions, since there are significantly more cells with nuclear blebs found on PLA than on the other substrates.

Cancer cells, abundant in the grooved nuclei, are in general associated with an acidic microenvironment. Thus, our identification of these structures on the PLA substrate might be a good starting point for further research of that phenomenon. For PHO, the number of the cells with grooves was similar to glass (reference), which may indicate that this could be a valuable material in terms of medical appliances. It has to be noted that PHO also undergoes hydrolysis in similar environments. Nevertheless, its products of degradation are far less acidogenic than lactic acid; PHO degrades to predominantly dimers of (*R*)-3-hydroxyacids and in a lower quantity to (*R*)-3-hydroxyoctanoic acid (pK_a_ = 4.84) [39]. Thus, the microenvironment of the cell is less affected by the pH change. The last question that can be raised in terms of the influence of the substrate on the creation of grooves and blebs in the nuclei is whether the expression of vimentin is notably altered depending on the type of the substrate. Do the microenvironmental features induce vimentin to support the creation of nuclear alterations? These questions remain to be answered.

## 4. Materials and Methods

### 4.1. Cell Culturing on Glass, PHO, and PLA

In the experiments, we cultured MEF 3T3 (mouse embryonic fibroblast) cells on three different substrates on dishes, under sterile conditions in an incubator that provided the stable temperature of 37 °C and level of carbon dioxide: 5% CO_2_. We used DMEM Low Glucose (Dulbecco’s Modified Eagle Medium, Biowest, Nuaillé, France), with 10% (*v*/*v*) FBS (Fetal Bovine Serum, Gibco Thermo Fisher Scientific, Waltham, MA, USA) and antibiotics 1% (*v*/*v*) of penicillin and 1% (*v*/*v*) (streptomycin, Sigma-Aldrich, Poznań, Poland) as a culture medium. The cells that were used in the experiments were between third and ninth passage, grown each time to approximately 80% of confluence until the passage procedure.

### 4.2. Cells Preparation and Staining

Cells were grown for 12 h on three different substrates: PHO (polyhydroxyoctanoate), PLA (polylactide), and glass as a reference. Then, cells were fixed in 4% formaldehyde solution in PBS, permeabilized with 0.1% (*v*/*v*) Triton-X solution in PBS and treated with 3% (*w*/*v*) BSA solution to block unspecific bonds (Sigma-Aldrich, Poznań, Poland). After that, overnight staining in 5 °C with Abcam Recombinant Anti-Vimentin antibody cytoskeleton marker ab92547 in BSA solution was performed, followed by 3-h staining of the nucleus with DAPI dye (Thermo Fisher Scientific Thermo Fisher Scientific, Waltham, MA, USA), and secondary staining of vimentin with Alexa Fluor 633 (Thermo Fisher Scientific) at room temperature (RT). After staining of vimentin and the nucleus, the staining of actin and/or microtubules was performed. Monoclonal Anti-α-Tubulin mouse antibody (Sigma-Aldrich, Poznań, Poland) was used in combination with Goat anti-Mouse IgG secondary antibody with Alexa Fluor 488 fluorophore (Thermo Fisher Scientific) (1 h in room temperature each). For staining actin, Alexa Fluor 488^®^ phalloidin was used (1 h in room temperature). The order of the staining was always as mentioned above: Anti-Vimentin Antibody (overnight), 3-h staining of DAPI alongside secondary vimentin staining with Alexa Fluor 633, and finally, 1-h staining of actin or 2-h staining of microtubules.

### 4.3. Preparation of PHO and PLA Substrates

PHO obtained for the substrates was prepared as described by Sofinska et al [20]. PLA used for the substrates was purchased from MG Chemicals (Burlington, ON, Canada) in a form of a string for 3D printing (PLA17TL1). Both PHO and PLA films were cast on a glass-bottom dish after the previous dissolution of the polymers in ethyl acetate in a proportion of 0.5 g of polymer per 10 mL of the solvent. The amount of 70 µL of the solution was used to cast a layer of a film per one glass-bottom dish. After that, films were left to dry for 48 h in a temperature of 50 ℃. Before cell culturing, the substrates were rinsed with sterile PBS twice. 

### 4.4. Microscopic Studies of Vimentin Cytoskeleton of MEF 3T3 Cells Grown on Different Substrates

The imaging of the stained cells was conducted on a Zeiss Axio Observer Z.1 microscope with LSM 710 confocal module. The analysis of the captured images was performed on Zeiss ZEN Black version 8.1.0.484 PALMRobo version V 4.6.0.4, (Carl Zeiss Microscopy GmbH Carl-Zeiss-Promenade 10 07745 Jena, Germany), and Fiji app (ImageJ 1.52p, Wayne Rasband National Institutes of Health, Bethesda, MA, USA) software (https://imagej.net/). The imaging was conducted on single cells with an oil immersion objective of 40× magnification, and numerical aperture (NA) of 1.4.

### 4.5. Statistical Analysis

A statistical analysis employing a *χ*^2^ test was performed in order to verify dependencies between the number of grooved nuclei and the substrate, the number of nuclei that were narrower at one end, and the number of nuclei with blebs. The differences were deemed statistically significant at probabilities of *χ*^2^_0.05_ and *χ*^2^_0.01_ (Origin Pro 2019 Software, OriginLab Corporation, Northampton, MA, USA). The experiment was performed 5 times for each substrate. Per sample, 15–25 cells were scanned.
